# Supplementary material for: A Platform of Patient-Derived Microtumors Identifies Individual Treatment Responses and Therapeutic Vulnerabilities in Ovarian Cancer
Source: Cancers (Basel). 2022 Jun 12;14(12):2895. doi: 10.3390/cancers14122895 (PMC9220902; doi:10.3390/cancers14122895)
Supplement: Supplementary file 1 [file cancers-14-02895-s001.zip › cancers-1713161-supplementary.pdf]

Supplementary Materials

A Platform of Patient-Derived Microtumors Identifies Individual Treatment Responses and Therapeutic Vulnerabilities in Ovarian Cancer

Nicole Anderle, André Koch, Berthold Gierke, Anna-Lena Keller, Annette Staebler, Andreas Hartkopf, Sara Y. Brucker, Michael Pawlak, Katja Schenke-Layland and Christian Schmees

SI Methods

Viability Measurement of OvCa PDM after Isolation

About n = 10 PDM were removed from PDM culture into a 96-well clear bottom plate and were stained with 2 µM viability dye Calcein-AM (Invitrogen) and 5 µM SYTOX orange nucleic acid stain (Invitrogen) in 200 µL culture media. After 30 min incubation time, z-stack images of stained PDM were taken in FITC and TRITC channel with a spinning disk microscope (ZEISS CellObserver Z1). 2D images of 3D z-stack projections within the Zen 2.6 software were generated. Percentages of PDM viability were assessed by using the Imaris 8.0 software. 3D surface masks were created for FITC and TRITC channel. Thresholds were adjusted and the sum of the total volume of each surface mask was measured. To calculate the percentage of viable/dead cells, the volume of each channel was divided by the total volume and multiplied by 100.

Histology/Immunohistochemistry

For histology of ovarian cancer microtumors, n = 5-10 PDM were fixed for 1 hour in 4% Roti® Histofix (Carl Roth) at RT and then collected in a 40 µm cell strainer. PDM were incubated for 5 min in Harris Hematoxylin (Leica Biosystems), shortly washed in dH_2O and dehydrated in an ethanol series (2x 50% ethanol, 2x 70% ethanol, each for 15 min). PDM were transferred into a Tissue-Tek® Cryomold® (Sakura) and embedded in Richard-Allan Scientific™ HistoGel™ (Thermo Fisher Scientific). After subsequent tissue processing with HistoCore PEARL (Leica Biosystems), PDM histogel-blocks were paraffin-embedded for sectioning. Three micrometer sections of FFPE PDM samples were prepared. Corresponding primary tumor tissue was received as cryosections (5-7 µm) from the Center for Women's Health, Tuebingen. Primary tissue samples (OvCa #24-26) were directly taken from the delivered tumor sample, fixed in 4% Roti® Histofix overnight at RT and paraffin-embedded after tissue processing. For Hematoxylin and Eosin (H&E) staining, PDM-FFPE sections were baked (60 min, 55°C), deparaffinized and rehydrated in decreasing ethanol series. Afterwards the sections were stained with Harris Hematoxylin (Leica Biosystems) for 2-3 min. Sections were washed in tap water for 3 min, incubated in 2% acetic acid for up to 2 min, neutralized in 95% ethanol, stained with Eosin Y (alcoholic, Leica Biosystems) for 2 min and at last dehydrated by an increasing ethanol series ending with Roti® Histol (Carl Roth). H&E staining of corresponding primary tumor tissue samples (cryosections) was performed within the pathology department of the Women's Health Clinic, Tuebingen.

For immunohistochemical staining of PDM-FFPE sections, antigen-retrieval was performed in TE buffer (10mM Tris/1mM EDTA, pH 9.0) at 95-98°C after slides were baked, deparaffinized and rehydrated in decreasing ethanol series to water. Cryosections of corresponding primary tumor tissue were simply rehydrated in PBS for 10 min. All sections (FFPE and cryosections) were subsequently incubated with BLOXALL® Endogenous Peroxidase and Alkaline Phosphatase Blocking Solution (VECTOR Laboratories) for 10 min. Slides were washed in PBS and incubated with blocking medium (PBST with 10% normal goat serum plus Novocastra™ Avidin) (Leica Biosystems) for 1 h at RT in a humidified

chamber. After two washing steps in PBS, sections were incubated with primary antibodies (see SI Materials) diluted in PBS, 1% BSA and Novocastra™ Biotin (Leica Biosystems) overnight at 4°C in a humidified chamber. Slides were washed 2x in PBS and incubated with secondary antibodies (see SI Materials) in PBS plus 1% BSA (30 min, at RT). Slides were washed in PBS and incubated with R.T.U Horseradish Peroxidase Streptavidin (VECTOR laboratories) for 30 min. Staining was accomplished by using the DAB (Polymer) Kit Novocastra Novolink™ (Leica Biosystems) for 5 min. Slides were counterstained with hematoxylin QS (VECTOR Laboratories). After dehydration and clearance in a rising ethanol series to Roti® Histol, the slides were mounted with CV mounting medium (Leica Biosystems). For high quality staining of p53 and WT1 in PDM, immunohistochemistry stainings were performed by the Institute of Pathology and Neuropathology at the University Hospital Tuebingen using routine and standardized immunohistochemistry protocols. Stained FFPE/cryosections were imaged with Axio Scan Z1. All primary antibodies were validated in normal, healthy tissues as well as in FFPE and cryosections.

DAB staining was quantified using ImageJ Fiji software. The percentage of area that is positive for DAB was determined. The color deconvolution plugin was used to separate stains using Ruifrok and Johnston's method described in [66]. Percent Area Fraction was measured as the percentage of pixels in the image or selection that have been thresholded.

Protein Profiling of PDM by RPPA

To generate protein abundance profiles of PDM and to analyze their dynamic on/off-target changes upon treatments, Reverse Phase Protein Array (RPPA) protein profiling using Zeptosens technology [15,16] was used. Cultured PDM (n = 100-200) were harvested, washed in HBSS, snap frozen in 0.65ml LoBind Eppis (Eppendorf) and stored at -80°C. To analyze treatment effects over time, four replicates of n = 25-35 PDM were treated with a drug, pooled, washed in HBSS and snap frozen after 30 min, 4 h and 72 h in 0.65 ml or 1.5 ml Protein LoBind tubes (Eppendorf) on dry ice. PDM pellets were stored at -80°C. For protein extraction from the low amount of PDM pellet, samples were lyophilized with a Epsilon 1-4 LSC plus instrument (Christ/Osterode). Lyophilisates were lysed with 6.25 µl CLB1 lysis buffer (Zeptosens) for 30 min (RT) at 1400 rpm (thermomixer) followed by centrifugation (5 min, 13200 rpm at RT). Protein amount was determined by Pierce™ Coomassie Plus™ (Bradford) Protein-Assay (Thermo Fisher Scientific). For printing, PDM lysates were adjusted to a uniform protein concentration (2 µg/µl) by diluting the samples in CLB1 if necessary, following vortexing, centrifugation (5 min, 13200 rpm, RT) and storage at -80°C. Adjusted PDM lysates were diluted 10-fold in RPPA spotting buffer CSBL1 and printed as replicate microarrays on Zeptosens hydrophobic chips (NMI TT) using a NanoPlotter 2 (GeSim), each sample at two technical replicates.. With the samples, fluorescence-labeled albumin (reference spots), internal standard lysates and further technical controls were co-printed for use as quality control. Freshly printed chips were blocked with 3% BSA solution, washed subsequently with dH_20, dried under a nitrogen stream and stored in the dark at 4°C.

Protein expression signals were measured using a direct two-step sequential fluorescence immunoassay. Up to six spotted chips (6x6 arrays) were assembled in a ZeptoCARRIER with fluidic structures. Well-characterized, pre-validated primary antibodies of interest (one antibody = one array) were incubated with the printed arrays at their pre-chosen dilution. (see SI Materials). Arrays were washed once with Zeptosens CAB1 assay buffer and incubated with primary antibodies (diluted in CAB1) over night at RT in the dark (15 h). Arrays were washed again once and incubated with Alexa647-labeled anti-species secondary antibodies (see SI Materials) for 45 min at RT in the dark. Arrays were flushed with assay buffer and imaged in the red laser of the ZeptoREADER imager system (Zeptosens). Typically, 6 images were taken at exposure times between 0.25 and 16 seconds. Non-specific assay signal contributions were evaluated from blank assays (arrays only incubated with secondary antibody). A separate protein stain assay was performed on one chip out of the print series to analyze printed protein per spot, used for normalizing the assay signals. Mean background-corrected spot signals from one image/exposure

time per assay - at highest signals below saturation - were quantified, using the ZeptoVIEW 3.1 array analysis software. Signals of the two replicate samples were averaged and normalized to printed protein (NFI = normalized mean fluorescence intensity). Array/assay quality and robustness were good as verified by replicate measurements of the standard assays (phospho-Erk1/2 and phospho-EGFR). NFI signals of the co-printed control and treatment standard lysates were quantified in two independent experimental runs, on two different arrays on different chips, at different times. NFI signals and TR (treatment-to-control ratios) are given in the Table below.

	p-EGFR (Tyr1068)			p-Erk1/2 (Thr202/Tyr204)		
	**control (NFI)**	**treatment (NFI)**	**TCR**	**control (NFI)**	**treatment (NFI)**	**TCR**
run1	0.078	0.334	4.30	8.648	19.768	2.29
run2	0.075	0.382	5.10	7.173	18.833	2.63
mean	0.076	0.358	4.70	7.911	19.300	2.46
CV	2.6%	9.5%	12.1%	13.2%	3.4%	9.8%

TR = treatment-to-control signal ratio

CVs of the NFI and of the calculated TR were in arrange below 10%. TR met their pre-defined quality criteria.

SI Materials

Immunohistochemistry primary antibodies, their source and the working concentrations.

Antibody	Species	Supplier	Dilution (IHC-P)	Dilution (IHC-Fr)	Control tissue
monoclonal anti-human Mesothelin (420404)	rat	RD Systemouse (MAB32651)	1:50	1:50	Tonsil
monoclonal anti-human CA-125 (MUC16) (X325)	mouse	Abcam (ab10033)	1:1000	1:1000	Bronchus
monoclonal anti-human PDL1 (E1L3N)	rabbit	Cell Signaling (13684)	1:200	1:200	Tonsil
monoclonal anti-human CD163 (D6U1J)	rabbit	Cell Signaling (93498)	1:500	1:500	Thymus
polyclonal anti-human Collagen alpha-1(I) chain	rabbit	NSJ Bioreagents (R31258)	1:1000	1:1000	Skin
polyclonal anti-human FAP alpha	rabbit	Bio-Rad Laboratories (AHP1322)	1:50	1:50	Bronchus
polyclonal anti-human C1QBP	rabbit	Sigma Aldrich (A63845)	1:50	1:50	Bronchus

Secondary antibodies used in Immunohistochemistry and RPPA.

Antibody	Conjugate	IgG-type	Species	Supplier	Dilution (IHC)	Dilution (RPPA)
anti-mouse IgG	Biotin-SP	F(ab')$_2$	Goat	Jackson ImmunoResearch (115-066-062)	1:1000	-
anti-rabbit IgG	Biotin-SP	IgG (H+L)	Goat	Jackson ImmunoReseach (111-065-144)	1:1000	-
anti-rat IgG	Biotin-SP	IgG (H+L)	Goat	Jackson ImmunoReseach (112-065-167)	1:1000	-
anti-goat IgG	Alexa647	F(ab')$_2$	Rabbit	Invitrogen (Z25608)	-	1:500
anti-mouse IgG	Alexa647	IgG (H+L)	Goat	Jackson ImmunoResearch (115-605-062)	-	1:3000
anti-rabbit IgG	Alexa647	IgG (H+L)	Goat	Invitrogen (A21245)	-	1:1000
anti-rat IgG	Alexa647	IgG (H+L)	Goat	Invitrogen (A21247)	-	1:1000

Antibodies used in protein quantification by RPPA.

Antigen	Modification-Site	Supplier	Product No. #	Species	Dilution	Uniprot (human)	#
4E-BP1		abcam (Epitom-ics)	ab32024	rabbit	200	Q13541	
4E-BP1 - phospho	Ser65	Cell Signaling	9456	rabbit	100	Q13541	
Actin beta		Cell Signaling	4970	rabbit	5000	P60709	
AFP		abcam (Epitom-ics)	ab52940	rabbit	200	P02771	
Akt		Cell Signaling	4685	rabbit	200	P31749	
Akt - phospho	Ser473	Cell Signaling	4060	rabbit	100	P31749	
Akt - phospho	Thr308	Cell Signaling	9275	rabbit	100	P31749	
Aurora A (AIK)		Cell Signaling	4718	rabbit	200	O14965	
Aurora A (AIK) - phos-pho	Thr288	Cell Signaling	3079	rabbit	100	O14965	
Aurora A/B/C - phos-pho	Thr288/Thr232/Thr 198	Cell Signaling	2914	rabbit	100	O14965, Q96GD4, Q9UQB9	
beta-Catenin		Millipore	06-734	rabbit	500	P35222	
beta-Catenin - delta		Cell Signaling	59854	rabbit	200	P30999	
beta-Catenin - phospho	Ser675	Cell Signaling	9567	rabbit	200	P35222	
beta-Catenin - phospho	Thr41/Ser45	Cell Signaling	9565	rabbit	100	P35222	
beta-Catenin - phospho	Ser45	Cell Signaling	9564	rabbit	100	P35222	
Caspase 3		Cell Signaling	9662	rabbit	200	P42574	
Caspase 3 - cleaved	Asp175	Cell Signaling	9661	rabbit	100	P42574	
Caspase 7 - cleaved	Asp198	abcam	ab2323	rabbit	100	P55210	
Caspase 8 - cleaved	Asp374/Asp391	Cell Signaling	9496	rabbit	100	Q14790	
CD137		abcam	ab209256	rabbit	200	Q07011	
CD163		abcam	ab199427	rabbit	200	Q86VB7	
CD44		abcam (Epitom-ics)	ab51037	rabbit	200	P16070	
CD44 variant (Exon v6)		Bender MedSys-temouse	BMOUSE125	mouse	200	P16070	
cdc2 (CDK1)		Cell Signaling	9112	rabbit	200	P06493	
cdc2 (CDK1) - phospho	Tyr15	Cell Signaling	4539	rabbit	200	P06493	
CDK2		Cell Signaling	2546	rabbit	200	P24941	
CDK2 - phospho	Thr160	Cell Signaling	2561	rabbit	100	P24941	
CDK4		Cell Signaling	12790	rabbit	200	P11802	
CDK4 - phospho	Thr172	Invitrogen	PA-64482	rabbit	100	P11802	
CDK6		Cell Signaling	13331	rabbit	200	Q00534	
CDK6 - phospho	Tyr24	biorabbityt	orabbit15014	rabbit	100	Q00534	
c-Jun		Cell Signaling	9165	rabbit	200	P05412	
c-Jun - phospho	Ser73	Cell Signaling	9164	rabbit	100	P05412	
c-Jun - phospho	Ser63	Cell Signaling	2361	rabbit	100	P05412	
c-Met (HGF/SF recep-tor) - phospho	Tyr1349	abcam (Epitom-ics)	ab68141	rabbit	100	P08581	
c-Raf		Cell Signaling	9422	rabbit	200	P04049	
c-Raf - phospho	Ser259	Cell Signaling	9421	rabbit	100	P04049	
Cyclin B1		abcam	ab32053	rabbit	200	P14635	
Cyclin B1 - phospho	Ser133	Cell Signaling	4133	rabbit	100	P14635	
Cyclin E2		abcam (Epitom-ics)	ab32103	rabbit	200	O96020	
E-Cadherin		R&D	AF748	goat	200	P12830	
EGFR (Erabbit-1, HER1)		Cell Signaling	4405	rabbit	200	P00533	
EGFR (Erabbit-1, HER1) - phospho	Tyr1068	Cell Signaling	2234	rabbit	100	P00533	

EGFR (Erabbit-1, HER1) - phospho	Tyr845	Cell Signaling	2231	rabbit	100	P00533
EGFR (Erabbit-1, HER1) - phospho	Tyr992	Cell Signaling	2235	rabbit	100	P00533
EGFR (Erabbit-1, HER1) - phospho	Tyr1068	Cell Signaling	2234	rabbit	100	P00533
EGFR (Erabbit-1, HER1) - phospho	Tyr1068	Cell Signaling	2234	rabbit	100	P00533
eIF2 alpha		Cell Signaling	9722	rabbit	200	P05198
eIF2 alpha - phospho	Ser51	Cell Signaling	3398	rabbit	100	P05198
eIF4E		Cell Signaling	2067	rabbit	200	P06730
eIF4E - phospho	Ser209	Cell Signaling	9741	rabbit	100	P06730
EpCAM (CD326)		Cell Signaling	3599	rabbit	200	P16422
Erk1/2 (MAPK p44/42)		Cell Signaling	4695	rabbit	200	P27361, P28482
Erk1/2 (MAPK p44/42) - phospho	Thr202/Tyr204	Cell Signaling	4370	rabbit	100	P27361, P28482
Erk1/2 (MAPK p44/42) - phospho	Thr202/Tyr204	Cell Signaling	4370	rabbit	100	P27361, P28482
Erk1/2 (MAPK p44/42) - phospho	Thr202/Tyr204	Cell Signaling	4370	rabbit	100	P27361, P28482
FAK1 - phospho	Tyr397	Cell Signaling	8556	rabbit	100	Q05397
FGF receptor 1		Cell Signaling	9740	rabbit	200	P11362
GSK3 alpha/beta - phospho	Ser21/Ser9	Cell Signaling	9331	rabbit	100	P49840 , P49841
GSK3 alpha/beta - phospho	Tyr279/Tyr216	abcam	ab68476	rabbit	100	P49840 , P49841
GSK3 beta		Cell Signaling	9315	rabbit	200	P49841
GSK3 beta - phospho	Ser9	Cell Signaling	9336	rabbit	100	P49841
Her2		Dako	A0485	rabbit	200	P04626
Her2 - phospho	Tyr1248	Cell Signaling	2247	rabbit	100	P04626
Histone H3		Cell Signaling	9715	rabbit	10000	P68431
Histone H3 - acetyl	Lys9/Lys14	Calbiochem	382158	rabbit	2000	P68431
Histone H3 - phospho	Ser10	Cell Signaling	9701	rabbit	100	P68431
Histone H3 - phospho	Thr11	Cell Signaling	9764	rabbit	100	P68431
Histone H3 - trimethyl	Lys27	Cell Signaling	9733	rabbit	100	P68431
IkappaB alpha		Cell Signaling	9242	rabbit	200	P25963
IkappaB alpha - phospho	Ser32	Cell Signaling	9241	rabbit	100	P25963
Ki-67		Dako	M7240	mouse	200	P46013
MEK1		Cell Signaling	9124	rabbit	200	Q02750
MEK1/2 - phospho	Ser217/Ser221	Cell Signaling	9154	rabbit	100	Q02750, P36507
MEK2		Cell Signaling	9125	rabbit	200	P36507
Mesothelin		R&D Systemouse	MAB32651	rat	200	Q13421
MKK4 (SEK1) - phospho	Ser257/Thr261	Cell Signaling	9156	rabbit	100	P45985
mTOR (FRAP)		Cell Signaling	2983	rabbit	200	P42345
mTOR (FRAP)- phospho	Ser2448	Cell Signaling	2971	rabbit	100	P42345
Nanog		Cell Signaling	4903	rabbit	200	Q9H9S0
N-Cadherin		BD Transduction	610920	mouse	200	P19022
NF-κB p65		abcam (Epitomics)	ab76311	rabbit	200	Q04206
NF-κB p65 - phospho	Ser536	Cell Signaling	3033	rabbit	100	Q04206
Oct-4		Cell Signaling	4286	mouse	200	Q01860

p27 (Kip1, CDKN1B) - phospho	Ser10	abcam (Epitomics)	ab62364	rabbit	100	P46527
p38 MAPK		Cell Signaling	9212	rabbit	200	Q16539
p38 MAPK - phospho	Thr180/Tyr182	Cell Signaling	9211	rabbit	100	Q16539
p53		Cell Signaling	2527	rabbit	200	P04637
p53 - acetyl	Lys382	Cell Signaling	2525	rabbit	100	P04637
p70 S6 Kinase		Cell Signaling	2708	rabbit	200	P23443
p70 S6 kinase - phospho	Thr421/Ser424	Cell Signaling	9204	rabbit	100	P23443
p70 S6 Kinase - phospho	Thr389	Cell Signaling	9205	rabbit	100	P23443
PARP		Cell Signaling	9532	rabbit	200	P09874
PARP - cleaved	Asp214	Cell Signaling	9541	rabbit	100	P09874
PCNA		Kremmer		rat	200	P12004
PD1		Cell Signaling	86163	rabbit	200	Q15116
PDGF receptor beta		Cell Signaling	3169	rabbit	200	P09619
PD-L1		Cell Signaling	13684	rabbit	200	Q9NZQ7
PI3-kinase p110 beta		Millipore	04-400	rabbit	200	P42338
PI3-kinase p85 alpha		abcam (Epitomics)	ab40755	rabbit	200	P27986
PP1 alpha - phospho	Thr320	abcam (Epitomics)	ab62334	rabbit	100	P62136
PTEN		Cell Signaling	9552	rabbit	200	P60484
PTEN - phospho	Ser380	Cell Signaling	9551	rabbit	100	P60484
Rad51		abcam (Epitomics)	ab109107	rabbit	200	Q06609
Rabbit		Cell Signaling	9313	rabbit	200	P06400
Rabbit - phospho	Ser807/Ser811	Cell Signaling	8516	rabbit	100	P06400
RSK 1 (p90RSK) - phospho	Ser380	Cell Signaling	9341	rabbit	100	Q15418
RSK 1 (p90RSK) - phospho	Thr573	abcam (Epitomics)	ab62324	rabbit	100	Q15418
RSK 1/2/3		Cell Signaling	9347	rabbit	200	Q15418, Q51812, Q15349
RSK 3 - phospho	Thr356/Ser360	Cell Signaling	9348	rabbit	100	Q15349
S6 ribosomal protein		Cell Signaling	2217	rabbit	200	P62753
S6 ribosomal protein - phospho	Ser240/Ser244	Cell Signaling	2215	rabbit	100	P62753
S6 ribosomal protein - phospho	Ser235/Ser236	Cell Signaling	2211	rabbit	100	P62753
Slug		Cell Signaling	9585	rabbit	200	O43623
Smad1		Cell Signaling	6944	rabbit	200	Q15797
Smad2 - phospho	Ser465/Ser467	Cell Signaling	3108	rabbit	100	Q15796
Smad2 - phospho	Ser245/Ser250/Ser255	Cell Signaling	3104	rabbit	100	Q15796
Smad2/3		Cell Signaling	3102	rabbit	200	Q15796, P84022
Snail		Cell Signaling	3879	rabbit	200	O95863
Src		Cell Signaling	2108	rabbit	200	P12931
Src - phospho	Tyr527	Cell Signaling	2105	rabbit	100	P12931
STAT 1		Cell Signaling	9175	rabbit	200	P42224
STAT 1 - phospho	Tyr701	Cell Signaling	9167	rabbit	100	P42224
STAT 3		Cell Signaling	4904	rabbit	200	P40763
STAT 3 - phospho	Ser727	Cell Signaling	9134	rabbit	100	P40763
STAT 3 - phospho	Tyr705	Cell Signaling	9145	rabbit	100	P40763
Tubulin acetylated		Sigma	T6793	mouse	200	P68366
WT1 (Wilms Tumor 1)		Cell Signaling	83535	rabbit	200	P19544

FACS antibodies, their source and the working concentrations.

Antibody	Species	Supplier	Staining	Panel	Dilution
anti-human CD3, FITC	mouse	BD Biosciences (345763)	extracellular	1, 2, 5	1:40
anti-human CD8, PerCP	mouse	Thermo Fisher Scientific (MHC0831)	extracellular	1, 2	1:40
anti-human CD8, FITC	mouse	BioLegend (301005)	extracellular	4, 7	1:20
anti-human CD4, PE-Cy7	mouse	BioLegend (344611)	extracellular	1, 3	1:20
anti-human CD137, PE	mouse	Miltenyi Biotec (130-098-878)	extracellular	2	1:11
anti-human CD137, BV421	mouse	BioLegend (309820)	extracellular	1	1:20
anti-human CD25, PerCP	mouse	BioLegend (356131)	extracellular	3	1:20
anti-human Foxp3, BV421	mouse	BioLegend (320123)	intracellular	3	1:20
anti-human CD39, PE-Dazzle 594	mouse	BioLegend (328223)	extracellular	4	1:20
anti-human PD1, BV421	mouse	BioLegend (329919)	extracellular	4, 7	1:20
anti-human CTLA4, PerCP-Cy5.5	mouse	BioLegend (369607)	extracellular	4	1:20

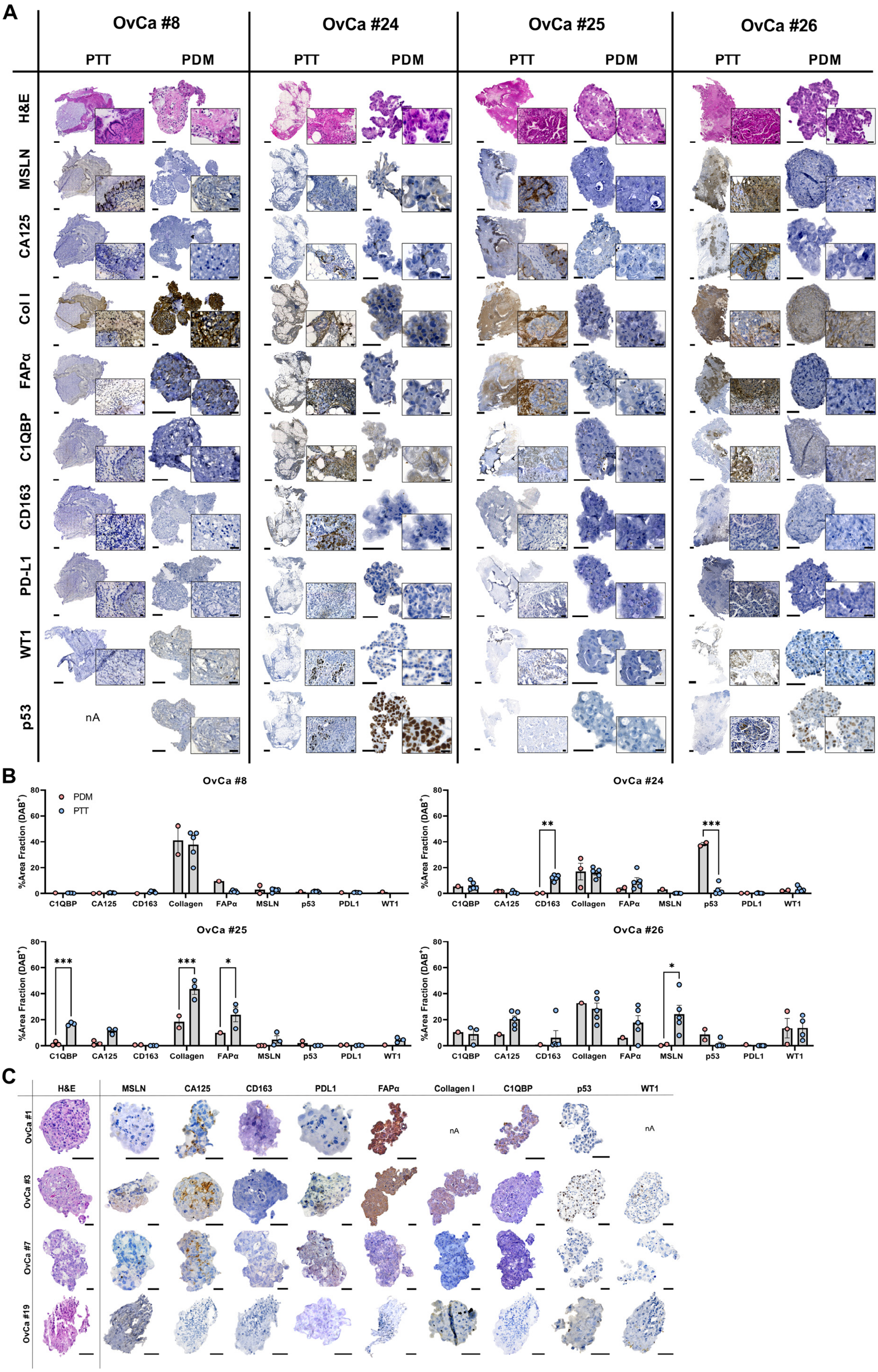

A
OvCa #8
OvCa #24
OvCa #25
OvCa #26
PTT
PDM
H&E
MSLN
CA125
Col I
FAPα
C1QBP
CD163
PD-L1
WT1
p53
nA
B
PDM
PTT
%Area Fraction (DAB+)
C1QBP
CA125
CD163
Collagen
FAPα
MSLN
p53
PDL1
WT1
C
H&E
Collagen I
OvCa #1
OvCa #3
OvCa #7
OvCa #19

Figure S1. Histology and immunohistochemistry of OvCa microtumors and corresponding PTT. (A) Characterization of OvCa microtumors and corresponding primary tumor tissue (PTT) by Hematoxylin & Eosin (H&E) staining and DAB immunohistochemical staining of OvCa clinical markers (p53, WT1), other cancer markers (CA125, mesothelin), tumor-associated macrophages CD163), immune/tumor marker (PD-L1), cancer-associated fibroblasts (FAPα) and extracellular matrix (Hyaloronan C1QBP, Collagen I). (**B**) Quantitative comparison of protein marker expression in OvCa PDM and corresponding PTT. Shown is the %Area Fraction of positive DAB-stain. For PTT a minimum of 3 representative regions of interest from tumor areas were used for quantification. (**C**) IHC staining of additional OvCa PDM models without available corresponding PTT sections. Scale bars indicate 500 µm for PTT; 50 µm for PDM; 20 µm for magnifications (PTT and PDM). Col I: Collagen I, MSLN: mesothelin.

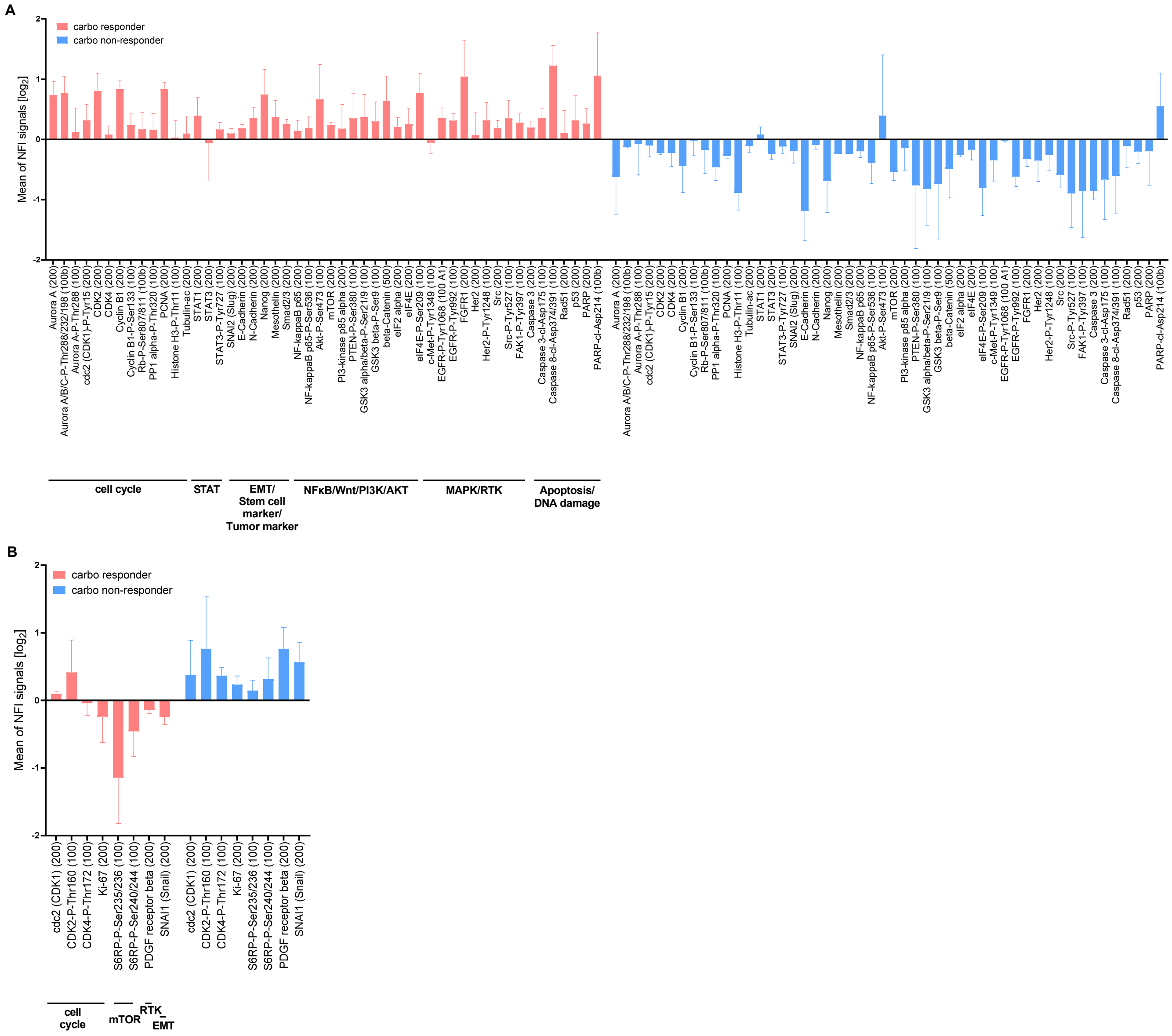

Figure S2. Up- and downregulated proteins in carboplatin responder and non-responder OvCa PDM. Mean protein abundances (NFI) within carboplatin responder and non-responder of untreated OvCa PDM. Proteins with >20% differential mean NFI signals between responder and non-responder were selected and plotted according to pathway affiliation. Data are shown as mean ± SEM (**A**) Proteins upregulated in carboplatin responder PDM. (**B**) Proteins downregulated in carboplatin responder PDM.

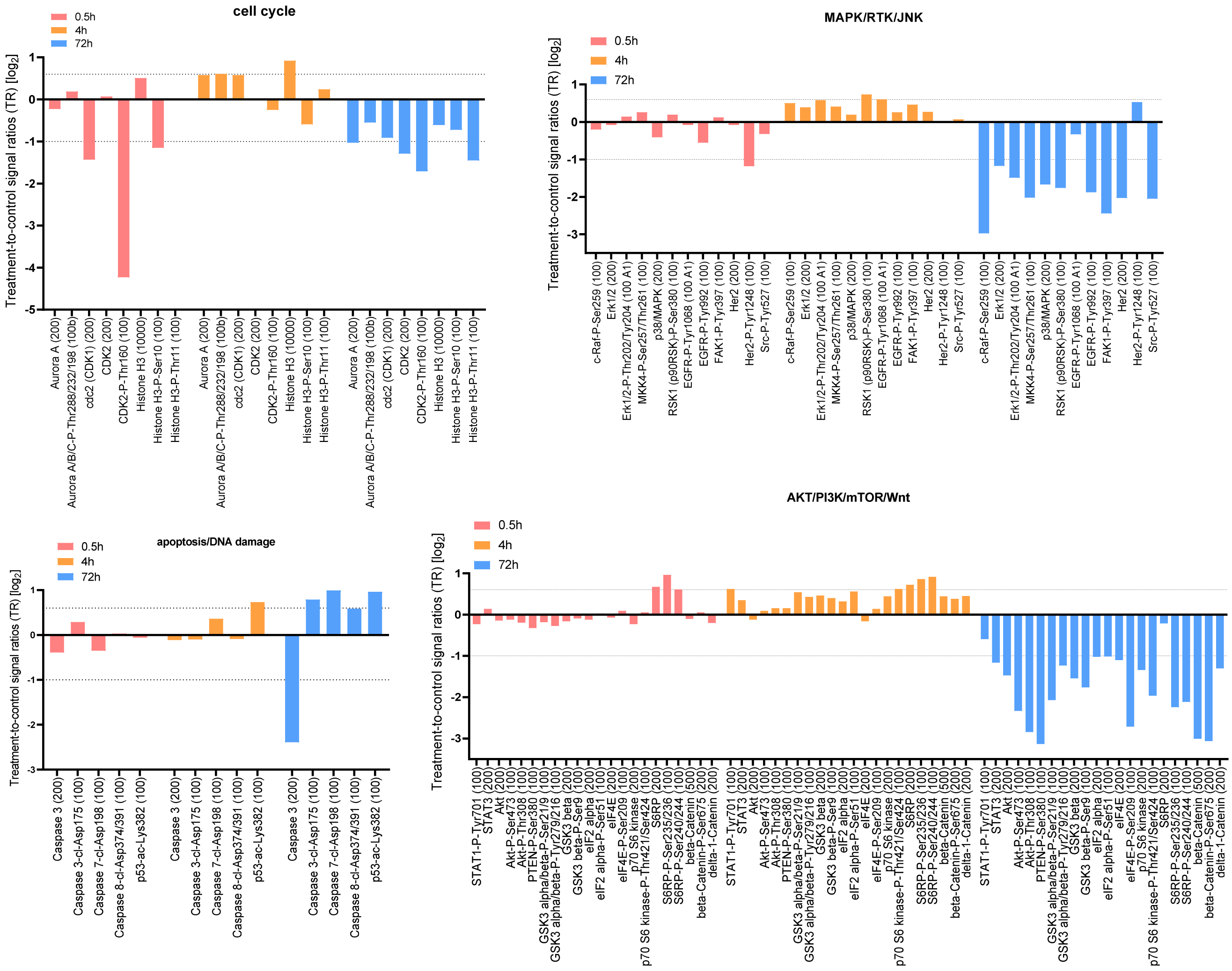

Figure S3. Time-dependent alterations of signaling pathways in carboplatin-sensitive OvCa PDM. On- and off-target effects of carboplatin treatment (75 µM) were assessed by RPPA analysis of treatment sensitive OvCa PDM. PDM from OvCa #24 were treated with carbo for different time spans to examine proteomic changes in the course of treatment. Protein abundances are displayed as treatment-to-control signal ratios (TR) calculated from NFI signals of treated PDM and DMSO vehicle control for each time point, log_2-transformed and sorted according to pathway affiliation. A threshold of >50% differential protein expression between treated samples and vehicle control was applied. Protein abundances are shown from 0.5 hours, 4 hours and 72 hours treated PDM. Carbo, carboplatin.

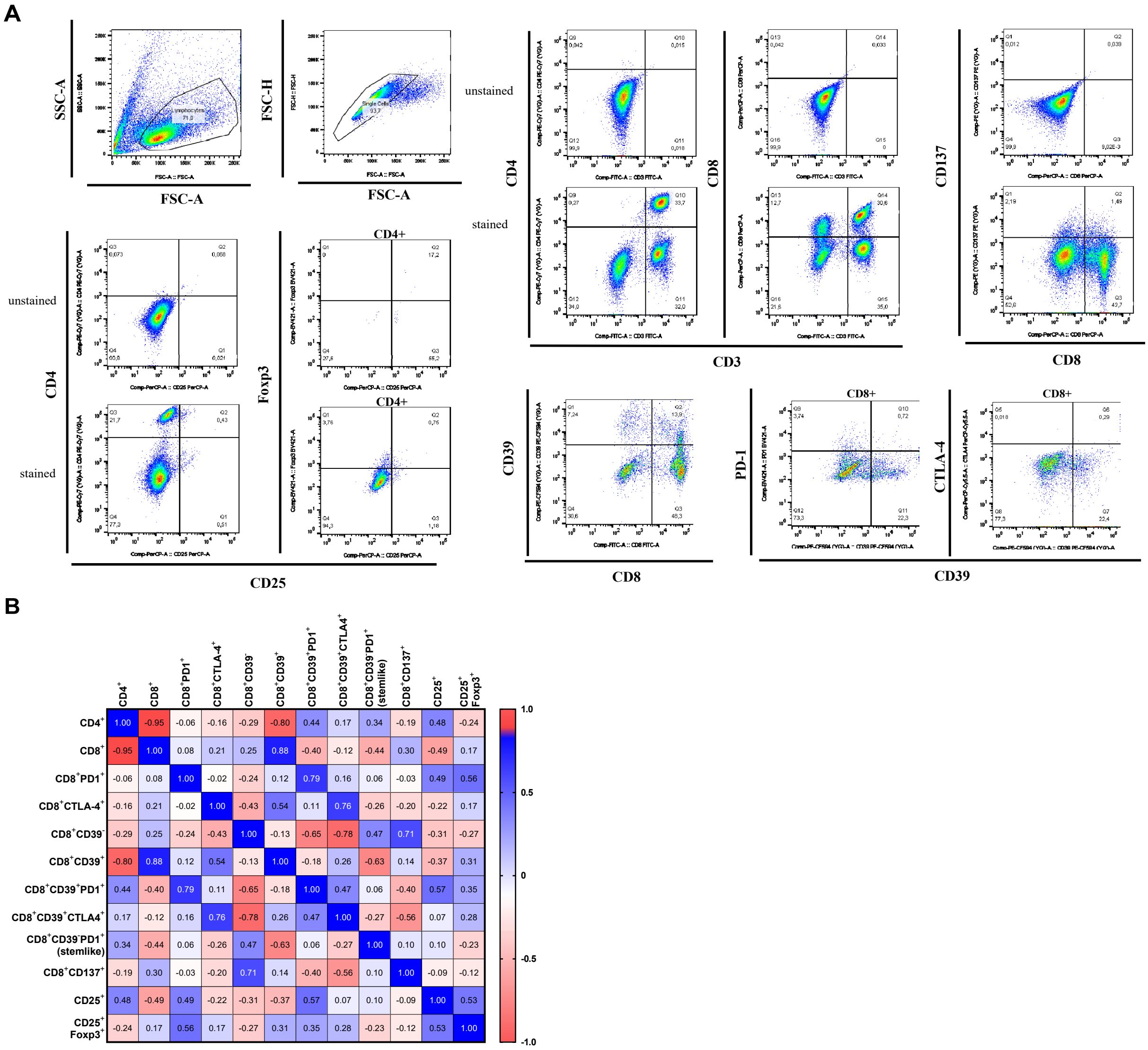

Figure S4. Gating schemes of expanded TILs, the correlation of TIL populations and their comparison between different OvCa models. (**A**) Exemplary FACS plots of multicolor stained OvCa TILs (OvCa #4) measured with FACS Melody (BD Biosciences) compared to unstained controls. (**B**) Nonparametric Spearman correlation of characterized TIL populations within n = 13 OvCa TIL samples. Shown are Spearman R-values. CTL, Cytotoxic lymphocytes.

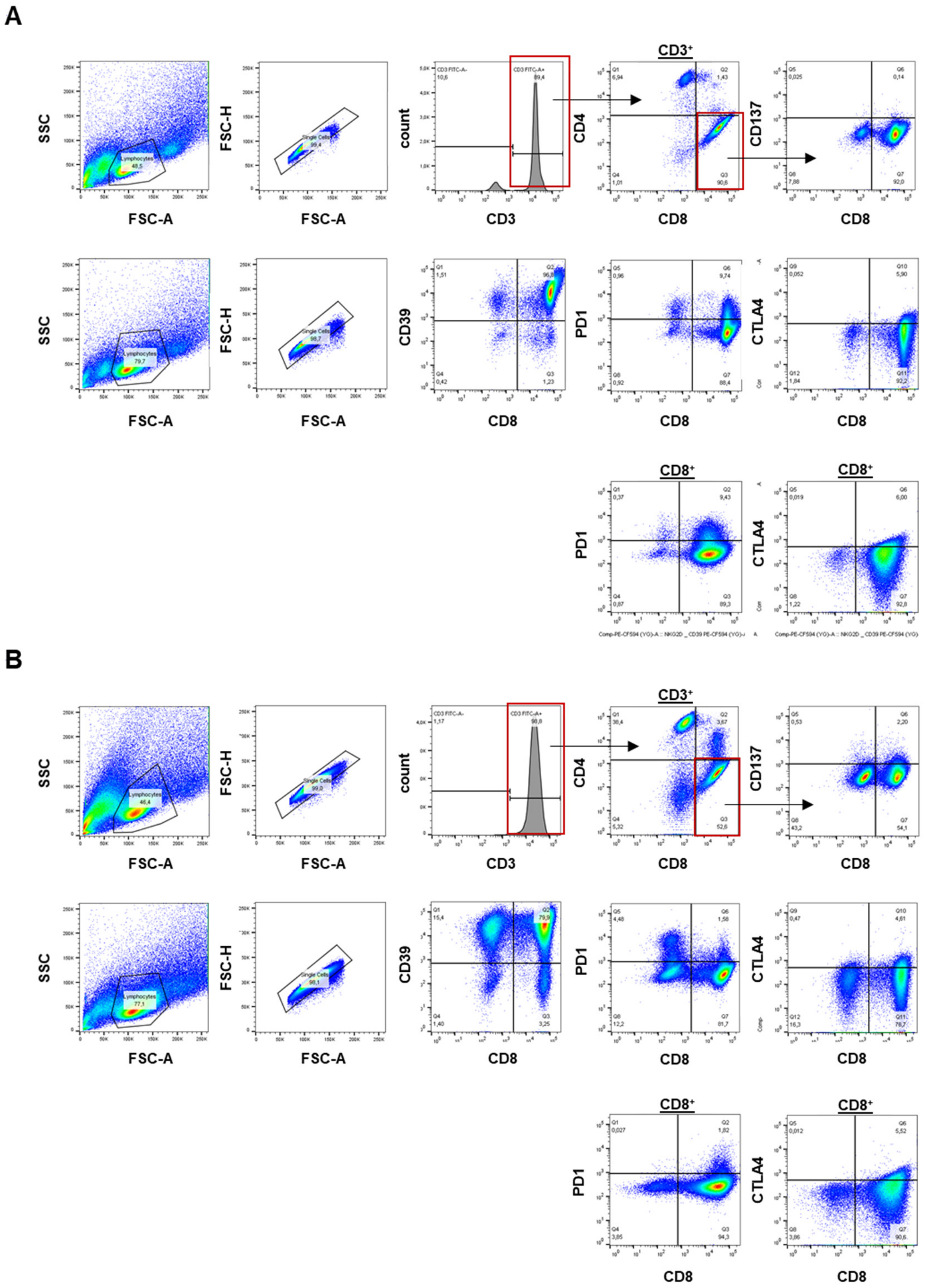

Figure S5. Gating schemes of expanded OvCa TILs #24 and #26. (**A**,**B**) FACS plots of multicolor stained (A) OvCa TILs #24 and (B) OvCa TILs #26 measured with FACS Melody (BD Biosciences). Shown are FACS plots of CD3$^+$CD8$^+$ and CD3$^+$CD4$^+$, CD8$^+$CD137$^+$, CD8+CD39$^+$, CD8+PD1$^+$, CD8$^+$CTLA4$^+$, CD8$^+$CD39$^+$PD1$^+$ and CD8$^+$CD39$^+$CTLA$^+$.

Table S1. Correlation of PDM isolation-success and clinical patient data (Spearman correlation).

	isolated PDM (yes/no) vs.				
	**Age OP**	**N (lymph node spread)**	**M (distant cancer spread)**	**Pn (perineural invasion)**	**FIGO stage**
**Spearman r**	-0.088	-0.386	-0.633	0.158	-0.166
**P (two-tailed)**	0.72	0.266	0.333	>.999	0.496
**P value summary**	ns	ns	ns	ns	ns
**Exact or approximate P value?**	Approxi-mate	Exact	Exact	Exact	Approximate
**Significant? (alpha = 0.05)**	No	No	No	No	No
**Number of XY Pairs**	19	13	6	13	19

Age was ranked into < 66 years = "0" and ≥ 66 years = "1"; N-M-Pn and PDM-isolation were classified as "yes" or "no".

Table S2. Log_2-transformed, median-centered NFI signals of signaling pathway proteins from OvCa and BC PDM from RPPA analysis.

	**Protein**	**OvCa #17**	**OvCa #19**	**OvCa #21**	**OvCa #23**	**OvCa #24**	**OvCa #25**	**OvCa #26**	**BC**
**cell cycle**	Aurora A (200)	0.86	0	-3.02	0.79	1.19	0.1	-1.24	-0.71
	Aurora A/B/C-P-Thr288/232/198 (100b)	1.24	-0.11	-0.63	0.86	0.99	-0.01	-0.14	0.5
	Aurora A-P-Thr288 (100)	1.05	-0.59	-0.37	-0.89	0	0.32	0.44	-0.09
	cdc2 (CDK1) (200)	0.16	0.89	-1.19	0.07	0.16	0	-0.13	-0.71
	cdc2 (CDK1)-P-Tyr15 (200)	0.88	0.09	-0.25	-0.27	0.63	0.03	-0.29	-0.04
	CDK2 (200)	0.75	-0.25	0.05	1.36	1.1	0	-0.2	-0.96
	CDK2-P-Thr160 (100)	1.14	1.53	-2.17	0.93	0.56	-0.97	0	-0.51
	CDK4 (200)	0.02	0	-0.54	0.24	0.36	-0.29	-0.45	0.1
	CDK4-P-Thr172 (100)	-0.39	0.24	0	-0.31	0.16	0.37	0.49	-0.22
	CDK6 (200)	0	0.21	0.17	-0.04	0.34	0.06	-0.34	-0.09
	CDK6-P-Tyr24 (100)	0.12	0.11	-0.04	-0.37	0.09	0	-0.21	0.08
	Cyclin B1 (200)	1.15	0	-1.68	0.44	0.94	0.81	-0.88	-0.66
	Cyclin B1-P-Ser133 (100)	0.57	0.22	-0.3	-0.24	0.5	0.11	-0.26	0
	Cyclin E2 (200)	0.02	0.13	-0.39	-0.13	0.08	-0.12	-0.19	0.01
	p27(Kip1)-P-Ser10 (100)	0.84	-0.5	-0.55	-1.2	-0.33	0.01	0	0.12
	Rb(CST9313 rb) (200b)	-0.32	0.22	0.35	0.62	0.31	0	-0.13	-0.43
	Rb-P-Ser807/811 (100b)	0.5	0.22	0.3	-0.53	0.7	0	-0.57	-0.31
	Ki-67 (200)	0	0.11	-3.86	-1.31	0.51	-0.16	0.36	0.52
	Histone H3-P-Ser10 (100)	1.36	1.22	-0.33	0.66	0.59	0	-0.16	-0.21
	Histone H3-P-Thr11 (100)	0.03	-1.17	0	-0.73	0.63	0.18	-0.61	0.81
	Tubulin-ac (200)	0.82	-0.22	0	-0.53	0.01	0.09	0	0.09
**MAPK/RTK**	c-Raf (200)	0.98	-0.87	-0.15	0.13	-0.14	0.02	0	-0.06
	Erk1/2 (200)	-0.6	0.33	1.53	2.47	0.59	0	-0.28	-0.72
	Erk1/2-P-Thr202/Tyr204 (100 A1)	0.49	-0.23	0	1.68	1.2	-0.63	0.37	-0.74
	MEK1 (200)	0.54	-0.23	-0.82	-0.32	0	0.26	0.33	-0.53
	MEK1/2-P-Ser217/221 (100)	0.7	0	-0.29	-0.8	0.2	-0.02	-0.04	0.28
	MEK2 (200)	-0.69	0	0.41	0.68	0.16	0.37	-0.08	-0.52
	MKK4-P-Ser257/Thr261 (100)	0.59	0	1.07	1.5	0.41	-0.13	-0.03	-0.7
	p38/MAPK (200)	-0.31	-0.32	2.33	1	0.06	0.3	0	-0.36
	p38/MAPK-P-Thr180/Tyr182 (100)	0.17	-0.35	0.47	0.26	0.3	0	-0.17	-0.59
	RSK1 (p90RSK)-P-Ser380 (100)	0.32	0	-0.1	1.29	0.95	-0.06	0.12	-0.6
	RSK1 (p90RSK)-P-Thr573 (100)	0.74	-0.03	-0.19	-0.03	-0.13	0.14	0.18	0
	RSK3-P-Thr356/360 (100)	-0.47	0	0.4	0.45	0.32	0.15	-0.22	-0.42
	c-Met-P-Tyr1349 (100)	0.28	-0.69	0.35	-0.55	0.06	0	0	-0.31
	EGFR (200)	0.7	0.27	-0.21	-0.1	0.36	0	-0.25	0.17
	EGFR-P-Tyr1068 (100 A1)	0.25	-0.04	0.24	-0.02	0.34	0.85	0	-0.69
	EGFR-P-Tyr845 (100)	-0.21	-0.06	0.35	0	0.17	0.5	0.21	-0.71
	EGFR-P-Tyr992 (100)	0.51	-0.78	1.53	0.43	0.31	0	-0.45	-0.1
	FGFR1 (200)	0.68	-0.2	1.05	2.79	0.69	0	-0.45	-0.52
	Her2 (200)	-0.84	0	0.03	0.23	0.96	-0.07	-0.7	0.15

	Her2-P-Tyr1248 (100)	0.88	0	-0.39	0.67	0.17	-0.45	-0.52	-1.08
	PDGF receptor beta (200)	-0.23	0.45	5.32	-0.14	0	-0.21	1.08	1.64
	Src (200)	0.04	-0.38	0.72	0.15	0.56	0	-0.79	-1.36
	Src-P-Tyr527 (100)	0.35	-1.46	2.2	0.38	1.07	-0.4	-0.33	0
	FAK1-P-Tyr397 (100)	0	-1.63	1.9	0.74	0.18	0.19	-0.08	-0.38
**PI3K/AKT/NFκB/Wnt**	IkappaB alpha-P-Ser32 (100)	0	0.4	0.3	0.4	0.48	-0.16	-0.65	-0.14
	NF-kappaB p65 (200)	0.65	-0.3	0.3	-0.16	0.08	0	-0.09	0
	NF-kappaB p65-P-Ser536 (100)	0.69	-0.73	-0.35	-0.19	0.21	0.04	-0.05	0
	Akt-P-Ser473 (100)	1.27	-0.61	1.4	-0.46	1.98	-0.13	1.4	-0.58
	Akt-P-Thr308 (100)	1.76	0	0.36	-0.59	2.96	-0.69	2.11	-0.45
	mTOR (200)	0.25	-0.68	0	0.24	0.35	0.13	-0.4	-1.07
	mTOR-P-Ser2448 (100)	-0.13	0.04	0.07	0	0.48	-0.08	-0.35	0.13
	PI3-kinase p110 beta (200)	0.49	0	-0.7	-0.27	0.17	0.08	-0.03	-0.18
	PI3-kinase p85 alpha (200)	1.18	-0.51	-0.07	-0.74	0	0.28	0.23	0
	PTEN-P-Ser380 (100)	-0.02	0.29	3.07	0	1.59	-0.17	-1.81	1.21
	GSK3 alpha/beta-P-Ser21/9 (100)	0	-1.43	0.7	0.31	1.43	-0.24	-0.21	0.22
	GSK3 beta-P-Ser9 (100)	-0.16	-1.65	0.56	0	1.25	0.1	0.18	-0.58
	beta-Catenin (500)	0.61	0	-0.83	1.68	0.58	-0.3	-0.97	0.02
**mTOR**	mTOR (200)	0.25	-0.68	0	0.24	0.35	0.13	-0.4	-1.07
	mTOR-P-Ser2448 (100)	-0.13	0.04	0.07	0	0.48	-0.08	-0.35	0.13
	4E-BP1-P-Ser65 (100)	0.58	-0.12	-0.42	-0.59	0.05	-0.02	0	0.17
	eIF2 alpha (200)	-0.1	-0.29	0.78	0.36	0.57	0	-0.22	0.94
	eIF4E (200)	0.19	-0.34	-0.19	-0.43	0.46	0.79	0	0.53
	eIF4E-P-Ser209 (100)	0.71	-1.26	0	-0.01	1.54	0.84	-0.34	1.94
	p70 S6 kinase-P-Thr389 (100)	0.14	0.12	-0.04	0.02	0.53	-0.1	0	-0.34
	p70 S6 kinase-P-Thr421/Ser424 (100)	-0.36	0.6	0.99	0.75	0.8	-0.34	0	-0.16
	S6RP-P-Ser235/236 (100)	-0.67	0.29	0.22	-2.42	0.54	-2.03	0	1.78
	S6RP-P-Ser240/244 (100)	-0.46	0.63	0.82	-1.07	0.59	-0.9	0	1.49
**apoptosis**	Caspase 3-cl-Asp175 (100)	0.15	-1.33	-1.49	0.04	0.49	0.75	0	-1.42
	Caspase 7-cl-Asp198 (100)	0.2	0	-0.53	0	0.07	2.98	1.37	-1.41
	Caspase 8-cl-Asp374/391 (100)	0.26	-1.22	-1.13	1.79	1.5	1.34	0	-2.37
	p53 (200)	0.09	0	0.73	1.49	0.09	-0.4	-0.4	-1.39
	p53-ac-Lys382 (100)	0.85	-0.47	-0.34	-0.5	0	0.56	0.62	-0.09
**tumor and stem cell marker**	p53 (200)	0.09	0	0.73	1.49	0.09	-0.4	-0.4	-1.39
	Smad2/3 (200)	0.31	-0.24	0	0.44	0.16	0.11	-0.24	-0.47
	Smad2-P-Ser245/250/255 (100)	-0.02	0.19	0.78	0.39	-0.16	0.33	0	-0.45
	Smad2-P-Ser465/467 (100)	0.33	0.17	-0.23	-0.39	0.14	-0.04	-0.09	0.22
	Nanog (200)	0	-1.21	-1.29	1.66	1.24	0.07	-0.16	-2.48
	OCT-4 (200)	1	-0.17	-0.19	-0.56	-0.1	0.72	0.49	0
	CD44v6 (200)	0.45	0	-0.08	-0.01	-0.36	0.61	0.53	-0.34
	Mesothelin (200)	0.04	-0.24	1.03	1.13	0.43	-0.12	-0.23	-0.56
	WT1 (Wilms Tumor 1) (200)	0.84	0.67	-0.87	0.6	-0.02	0	0.16	-1.95

Table S3. Spearman correlation of carboplatin-treatment sensitivity and protein abundances of OvCa PDM models.

Treatment sensitivity	Protein	Spearman r	P (two-tailed)	Exact P value	Significant (alpha = 0.05)	Number of XY Pairs
Carboplatin (75-125uM) vs.	Aurora A/B/C-P-Thr288/232/198 (100b)	0.8827	0.044	*	Yes	6
	Cyclin B1 (200)	0.971	0.011	*	Yes	6
	PP1 alpha-P-Thr320 (100)	0.8827	0.044	*	Yes	6
	PCNA (200)	0.8827	0.044	*	Yes	6
	PDGF receptor beta (200)	-0.8827	0.044	*	Yes	6
	NF-kappaB p65 (200)	0.8827	0.044	*	Yes	6
	NF-kappaB p65-P-Ser536 (100)	0.8827	0.044	*	Yes	6

Table S4. Significant difference in metastasis-free-survival between OvCa PDM carboplatin responder and non-responder "1": Event occurred; "0": censored.

OvCa #	Time (days)	Time (months)	Carboplatin responder	Carboplatin non-responder
17	494	16.2	1	
23	444	14.6	0	
24	293	9.6	1	
25	414	13.6	0	
19	281	9.2		1
26	4	0.1		0

Table S5. Descriptive statistics of analyzed OvCa TIL populations.

	$CD4^+$	$CD8^+$	$CD137^+$	$PD1^+$	$CTLA\text{-}4^+$	$CD39^-$	$CD39^+$	$CD39^+ PD1^+$	$CD39^+ CTLA4^+$	$CD39^+ PD1^+$	$CD39^- PD1^+$	CD25+ Foxp3+
**Number of values**	13	13	13	13	13	13	13	13	13	13	13	13
**Minimum**	6.94	4.21	0.140	1.48	0.00	0.00	4.36	0.720	0.00	0.720	0.00	0.00
**Maximum**	95.5	90.6	10.2	20.1	10.1	48.3	96.8	54.5	22.7	54.5	50.5	62.2
**Range**	88.6	86.4	10.1	18.6	10.1	48.3	92.4	53.8	22.7	53.8	50.5	62.2
**Mean**	57.8	33.5	3.09	6.88	3.44	9.49	40.5	15.7	5.41	15.7	7.31	7.45
**Std. Deviation**	26.9	23.5	3.18	6.35	3.13	13.2	29.4	18.0	6.19	18.0	15.2	16.9
**Std. Error of Mean**	7.45	6.52	0.881	1.76	0.868	3.66	8.15	4.98	1.72	4.98	4.22	4.68
**Lower 95% CI of mean**	41.6	19.3	1.17	3.04	1.54	1.52	22.7	4.82	1.67	4.82	-1.88	-2.74
**Upper 95% CI of mean**	74.1	47.7	5.01	10.7	5.33	17.5	58.3	26.5	9.15	26.5	16.5	17.6
**Coefficient of variation**	46.4%	70.1%	103%	92.3%	91.1%	139%	72.6%	115%	114%	115%	208%	226%

Table S6. Correlation of TIL phenotypes (Spearman correlation; P-Values).

	**$CD4^+$**	**$CD8^+$**	**$CD8^+PD1^+$**	**$CD8^+CTLA\text{-}4^+$**	**$CD8^+CD39^-$**	**$CD8^+CD39^+$**	**$CD8^+CD39^+PD1^+$**	**$CD8^+CD39^+CTLA4^+$**	**$CD8^+CD39^-PD1^+$ (stemlike)**	**$CD8^+CD137^+$**	**$CD25^+Foxp3^+$**
**$CD4^+$**	X	**0.000**	0.845	0.591	0.344	**0.002**	0.135	0.581	0.248	0.529	0.437
**$CD8^+$**	**0.000**	X	0.803	0.493	0.404	**0.000**	0.176	0.686	0.138	0.325	0.579
**$CD8^+PD1^+$**	0.845	0.803	X	0.960	0.417	0.699	**0.002**	0.605	0.839	0.924	0.050
**$CD8^+CTLA\text{-}4^+$**	0.591	0.493	0.960	X	0.140	0.058	0.723	**0.003**	0.385	0.505	0.579
**$CD8^+CD39^-$**	0.344	0.404	0.417	0.140	X	0.669	**0.018**	**0.003**	0.110	**0.009**	0.373
**$CD8^+CD39^+$**	**0.002**	**0.000**	0.699	0.058	0.669	X	0.566	0.396	**0.024**	0.643	0.297
**$CD8^+CD39^+PD1^+$**	0.135	0.176	**0.002**	0.723	**0.018**	0.566	X	0.106	0.845	0.182	0.239
**$CD8^+CD39^+CTLA4^+$**	0.581	0.686	0.605	**0.003**	**0.003**	0.396	0.106	X	0.370	**0.048**	0.355
**$CD8^+CD39^-PD1^+$ (stemlike)**	0.248	0.138	0.839	0.385	0.110	**0.024**	0.845	0.370	X	0.733	0.438
**$CD8^+CD137^+$**	0.529	0.325	0.924	0.505	**0.009**	0.643	0.182	**0.048**	0.733	X	0.696
**$CD25^+Foxp3^+$**	0.437	0.579	0.050	0.579	0.373	0.297	0.239	0.355	0.438	0.696	X

SI References

15. Kresbach, G.M.; Pawlak, M. High precision rppa: Concept, features, and application performance of the integrated zeptosens platform. *Adv. Exp. Med. Biol.* **2019**, *1188*, 31-59.

16. Pawlak, M.; Schick, E.; Bopp, M.A.; Schneider, M.J.; Oroszlan, P.; Ehrat, M. Zeptosens' protein microarrays: A novel high performance microarray platform for low abundance protein analysis. *Proteomics* **2002**, *2*, 383-393.

66. Ruifrok, A. C. & Johnston, D. A. Quantification of histochemical staining by color deconvolution. Anal. Quant. Cytol. Histol. 23, 291-299 (2001).
